# Exosome-Mediated Immunosuppression in Tumor Microenvironments

**DOI:** 10.3390/cells11121946

**Published:** 2022-06-16

**Authors:** Qi-Hui Xie, Ji-Qi Zheng, Jia-Yi Ding, Yu-Fei Wu, Luisa Liu, Zi-Li Yu, Gang Chen

**Affiliations:** 1 The State Key Laboratory Breeding Base of Basic Science of Stomatology (Hubei-MOST) & Key Laboratory of Oral Biomedicine Ministry of Education, School and Hospital of Stomatology, Wuhan University, Wuhan 430079, China; xieqihui@whu.edu.cn (Q.-H.X.); jiqizheng@whu.edu.cn (J.-Q.Z.); dingjiayi@whu.edu.cn (J.-Y.D.); wuyufei@whu.edu.cn (Y.-F.W.); luisaliu528@whu.edu.cn (L.L.); 2 Department of Oral and Maxillofacial Surgery, School and Hospital of Stomatology, Wuhan University, Wuhan 430079, China; 3 Frontier Science Center for Immunology and Metabolism, Wuhan University, Wuhan 430071, China

**Keywords:** exosomes, tumor microenvironments, immunosuppression, tumor cell, immune cell

## Abstract

Exosomes are membranous structures secreted by nearly all cell types. As critical messengers for intercellular communication, exosomes deliver bioactive cargoes to recipient cells and are involved in multiple physiopathological processes, including immunoregulation. Our pioneering study revealed that cancer cells release programmed death-ligand 1-positive exosomes into the circulation to counter antitumor immunity systemically via T cells. Tumor cell-derived exosomes (TDEs) also play an immunosuppressive role in other immunocytes, including dendritic cells (DCs), macrophages, natural killer (NK) cells, and myeloid-derived suppressor cells (MDSCs). Moreover, exosomes secreted by nontumor cells in the tumor microenvironments (TMEs) also exert immunosuppressive effects. This review systematically provides a summary of the immunosuppression induced by exosomes in tumor microenvironments, which modulates tumor growth, invasion, metastasis, and immunotherapeutic resistance. Additionally, therapeutic strategies targeting the molecular mechanism of exosome-mediated tumor development, which may help overcome several obstacles, such as immune tolerance in oncotherapy, are also discussed. Detailed knowledge of the specific functions of exosomes in antitumor immunity may contribute to the development of innovative treatments.

## 1. Introduction

Extracellular vesicles (EVs), membranous structures secreted by nearly all cell types, consist of microparticles and exosomes [1]. The classification of EVs is based on the differences in formation mechanism and size. Microparticles are secreted by cells through direct membrane budding, whereas exosomes are secreted through the endosomal transport pathway. According to the guideline recommended by the International Society for Extracellular Vesicles (ISEV) in 2018, EVs are divided into small EVs (sEVs, <200 nm) and large EVs (lEVs, >200 nm). Additionally, sEVs are mainly composed of exosomes and a small amount of microparticles that are less than 200 nm [2]. Exosomes inherit bioactive molecules from their parental cells, including nucleotides, proteins, and lipids. The crucial role of exosomes in intercellular communication has been widely recognized in the last 10 years, although they were once thought to be the excretion of cellular wastes for cell homeostasis. Numerous studies on targeted and functional interactions between exosomes and cells have revealed the significant biological functions of exosomes. Exosomes are involved in various physiopathological processes, such as embryonic development, tissue repair and regeneration, material metabolism, and immunoregulation [3]. Specially, exosomes play a critical role in regulating tumorigenesis and tumor progression. Accumulating studies have reported the tumorigenic effect of exosomes, especially in resistance to therapy [4]. A deeper investigation of exosomes in the disease state may contribute to a better understanding of the pathogenic mechanisms and help to develop innovative diagnostic or therapeutic strategies. This review summarizes the immunosuppressive effects of exosomes and discusses the potential clinical applications of exosomes in the diagnosis and treatment of tumor.

## 2. Life Course of Exosomes

**Figure 1:**
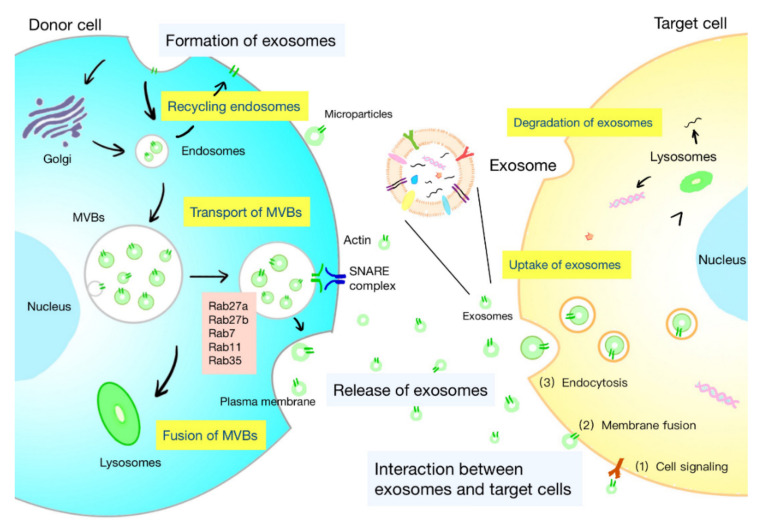
Life course of exosomes. The formation of exosomes: initiation, endocytosis, multivesicular bodies (MVBs) formation, and secretion. The intracellular trafficking of MVBs is mediated by Rab GTPases. The fusion of MVBs with the plasma membrane is facilitated by SNAREs. There are three kinds of interactions between exosomes and cells: (1) the membrane proteins on the exosomes and target cells bind directly, and then trigger the intracellular signaling cascade; (2) exosomes transport their contents to target cells by fusing with the cell membrane; and (3) exosomes are engulfed by cells and degraded by lysosomes to release signal molecules.

The life course of exosomes comprises the generation, secretion, transport of exosomes, and interaction with target cells, which is under the control of a series of complex regulatory molecules (Figure 1). A deeper understanding of the life course of exosomes is helpful to identify the functions of exosomes and develop strategies for specific regulation of exosomes.

### 2.1. Formation of Exosomes

The biogenesis of exosomes is a strictly regulated process that comprises four main stages: initiation, endocytosis, multivesicular bodies (MVBs) formation, and secretion (Figure 1) [5]. The generation of exosomes is mostly dependent on the endosomal sorting complex required for transport (ESCRT), which is composed of four complexes: ESCRT-0 (Hrs, Stam1, Stam2), ESCRT-I (Vps28, Vps37, Tsg101, etc.), ESCRT-II (Vps25, Vps36, etc.), and ESCRT-III (Vps2, Alix, etc.). It can recognize ubiquitinated proteins in vivo and assist in the transport of proteins and release of exosomes. The formation of MVBs is caused by an inward budding of the early endosomal membrane that is triggered by ceramide [6]. MVBs are normally degraded by fusion with lysosomes, while some are secreted into the extracellular space by fusion with the plasma membrane, which are called exosomes.

### 2.2. Release of Exosomes

Exosomes are released into the extracellular space through the fusion of the MVB limiting membrane with the plasma membrane (Figure 1). Rab GTPases mediate intracellular trafficking of MVBs and determine the fate of MVBs. Several Rab proteins, including Rab7, Rab11, Rab27, and Rab35, participate in exosome biogenesis. The release of flotillin-enriched exosomes and the transport of cargoes, such as transferrin receptors and signaling molecules, are reported to be regulated by Rab11 and Rab35 [1,7]. Certain membrane lipids are also found to participate in the secretion of exosomes. Phosphatidic acid originating from the activity of diacylglycerol kinase (DGK) or phospholipase D influences the secretion of exosomes [8,9]. The effects of the ethylmaleimide-sensitive factor attachment protein receptor (SNARE) in exosome secretion have been proved [10]. The fusion of MVBs with the plasma membrane is facilitated by the interaction between vesicular soluble SNAREs (v-SNAREs) on MVBs and target SNAREs (t-SNAREs) on the target membrane.

### 2.3. Interaction between Exosomes and Target Cells

After being released, exosomes are widely distributed to the blood, saliva, urine, cerebrospinal fluid, ascites, and pleural fluid. Exosomes can interact with both adjacent and distant cells to affect their cell activity and functions. Understanding the interplay between exosomes and target cells may help interpret the influences of exosomes on target cells. The interaction between exosomes and cells can be summarized as follows (Figure 1): (1) the membranal proteins on the exosomes and target cells bind directly, and then trigger the intracellular signaling cascade in target cells; (2) exosomes transport their contents to target cells by fusing with the cell membrane; and (3) exosomes are engulfed by cells and degraded by lysosomes to release signal molecules [5]. Besides, some mediators of these interactions have been found, including integrins, lipids, tetrapeptide, heparan sulfate proteoglycans, and extracellular matrix components [1]. The internalization of exosomes is suggested to be the primary interaction between exosomes and target cells. Exosomes are internalized through the endocytic pathway and transported to lysosomes, where the proteins and lipids in the exosomes are degraded to offer relevant metabolite sources to target cells [11]. Studies have shown the restricted colocalization of exosomes with early endosomes. It is believed that intraluminal vesicles could transfer contents to recipient cells by fusion with MVBs instead of lysosome [1].

## 3. Immunosuppressive Role of Tumor Cell-Derived Exosomes

**Figure 2:**
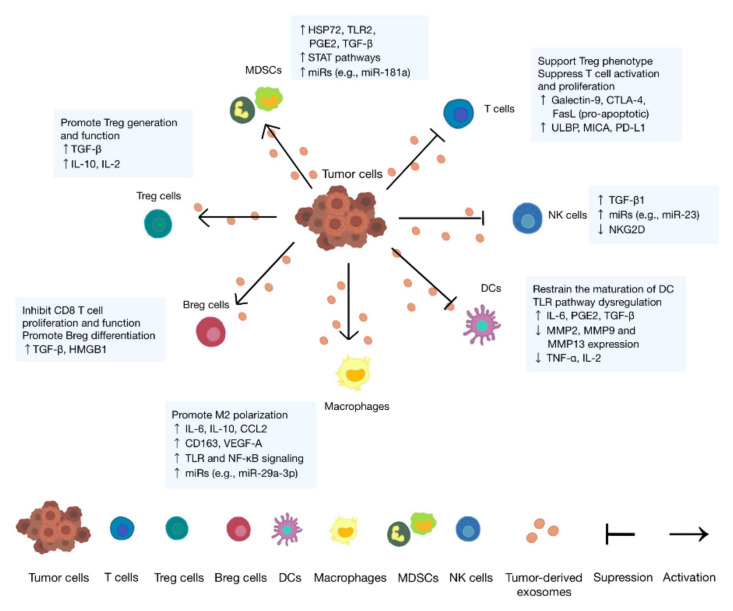
Tumor-derived exosomes contribute to the formation of the immunosuppressive tumor microenvironment. Tumor-derived exosomes can either suppress immune cells or contribute to the activation of immune cells. Suppression: induce T cells’ apoptosis and suppress their activation and proliferation, inhibit the differentiation and maturation of DCs, and suppress the immunity ability of NK cells. Activation: promote the proliferation and function of regulatory T and B cells (Tregs and Bregs), the polarization of M2 macrophages, and the function and expansion of MDSCs.

Normally, T cells accept tumor-associated antigens (TAAs) presented by antigen-presenting cells (APCs) and exert cytotoxicity against tumor cells. Besides, NK cells exert cytotoxicity by directly recognizing specific signal molecules on tumor cells. Studies have found that tumor cells escape immunity by reducing immunogenicity, inducing suppressor cells, modulating antigen presentation, and secreting immunosuppressive factors [12]. TDEs are confirmed to be involved in the complex network and contribute to the formation of the immunosuppressive microenvironment (Figure 2) and thereby promote tumor progression [13]. In the following paragraphs, we will focus on the immunosuppressive effects of TDEs on immune cells, such as T cells, dendritic cells (DCs), macrophages, NK cells, and MDSCs, and simply list them in a table (Table 1).

### 3.1. Lymphocytes

Lymphocytes, including T cells, B cells, and NK cells, are the main executors of immune functions, and play significant roles in the host immune response. They also work as a frontline “soldier” to fight infection and monitor cell variation. T cells originate from bone marrow progenitors and mature in the thymus and finally export to the periphery [41]. Different from T cells, both the differentiation and maturation of B cells occur in the bone marrow [42]. After activation, lymphocytes differentiate into effective cells to inhibit tumors. The activation of lymphocytes is modulated by a series of signal molecules. Negative immune checkpoints, such as programmed cell death receptor-1 (PD-1), cytotoxic T-lymphocyte antigen-4 (CTLA-4), and T cell immunoglobulin-3 (TIM-3) on immune cells, can induce cell exhaustion [43,44]. Exhaustion of T cells is found to be related to the poor outcome of tumor patients [45,46,47,48]. Tumor cells can express corresponding ligands, such as programmed death-ligand 1 (PD-L1), to exert immunosuppressive function [49].

CD4 and CD8 T cells play important roles in the specific antitumor immunity. Emerging pieces of evidence indicate that TDEs could carry various immunosuppressive signals to inhibit T cell proliferation and function in tumor immunity. The Fas/FasL signaling pathway is a significant regulator of T cell apoptosis. Studies have suggested that TDEs could express membrane-formed FasL to induce T cell apoptosis in a selective manner or suppress T cell receptor signal by decreasing the expression of CD3-ζ [15]. More recent studies have found the high level of exosomal PD-L1, which has the same topology as cell PD-L1. Exosomal PD-L1 is reported to interact with PD-1 on CD8 T cells, inducing inactivation of T cells and immune escape of tumor cells [16]. The proliferation and function of effector T cells treated with exosomal PD-L1 is inhibited, with decreased expression of CD69 and decreased secretion of cytokines, such as interferon-gamma (IFN-γ), tumor necrosis factor-alpha (TNF-α), and interleukin-2 (IL-2) [16]. As previously reported, galectin-9 in the exosomes from nose pharynx cancer cells induced the apoptosis of massive Epstein–Barr virus-specific CD4 T cells and inhibited the function of T helper 1 (Th1) cells [14]. UL16-binding proteins (ULBP) and major histocompatibility complex class I chain-related protein A (MICA) delivered by exosomes could inhibit the natural killer group 2 member D (NKG2D) signaling pathway, which is essential for the cell killing function of T cells [17]. In addition, exosomes with transforming growth factor-beta (TGF-β) could reduce the expression of NKG2D on CD8 T cells and prevent the activation of cells [18].

Apart from directly affecting CD4 and CD8 T cells, TDEs can induce immune suppression through inhibition of Th cells. Studies have found that circulating exosomes in patients with nasopharyngeal cancer carry abundant miR-24-3p, which impedes the differentiation of Th1 and Th17 cells through the repression of fibroblast growth factor 11 [19]. By means of releasing immunosuppressive cytokines, such as TGF-β and IL-10, to suppress antigen-specific CD8 T cells, Tregs can protect tumor cells from immune system attack [50]. Meanwhile, enhanced expression of CD25 and α-chain of IL-2R on Tregs can competitively bind IL-2, the critical element for the proliferation and activation of CD8 T cells, to inhibit the activation, proliferation, and antitumor ability of CD8 T cells [51]. TDEs have been demonstrated to facilitate the generation and expansion of Tregs. In colorectal cancer, via the activation of TGF-β/Smad signaling and devitalization of SAPK signaling, TDEs rich in TGF-β upregulate Treg-related genes [20]. Additionally, TDEs employ IL-10 or skew IL-2 to increase the amount of Tregs and promote their function [33].

B cells also play significant roles in host immunity, such as producing immunoglobulins, presenting antigens, providing costimulatory signals, and releasing cytokines to modulate antitumor immunity [18]. Bregs, a subgroup of B cells, can inhibit adaptive immunity by secreting inhibitory cytokines, such as IL-10, IL-21, IL-35, and TGF-β1, or expressing negative immune checkpoints, such as PD-L1 [23]. The generation of Tregs and the function of MDSCs and CD4 T cells are also under the regulation of Bregs [15]. Many studies have shown the influence of TDEs on the proliferation and function of Bregs. According to a study, exosomes derived from esophageal cancer cells promoted the differentiation of naïve B cells into TGF-β-producing Bregs, which exacted immunosuppressive effects on the proliferation of CD8 T cells [22]. Besides, TDEs carrying high mobility group protein B1 (HMGB1) are reported to induce the differentiation of T cell immunoglobulin and mucin domain-1 (TIM-1)^+^ Bregs to inhibit the cytotoxicity of CD8 T cells and promote survival and metastasis of tumors [23].

### 3.2. Macrophages

Macrophages are the main effector cells in innate immunity. They originate from the mononuclear phagocyte immune system. Macrophages play significant roles in anti-infective immunity. With the intention of fighting infection and maintaining tissue homeostasis, they will engulf and digest foreign and harmful substances, such as tumor cells and cellular debris [36]. Aside from stimulating the immune system, macrophages also play roles in modulating immunity by secreting diverse cytokines and by activating the complement system, which is likely to bring about inflammation [5,15]. The classical activation of macrophages is induced by a couple of cytokines, such as lipopolysaccharide and IFN-γ, while alternative activation is induced by cytokines such as IL-4 and oxidized lipids [52]. Consequently, macrophages can be polarized into two types: classically activated macrophages (M1) and alternatively activated macrophages (M2). These two types of macrophages have distinct functions in immune defense and immune surveillance [53]. With the internal environment changing, the two types of macrophages can transform into each other. M1 macrophages play a significant role in antitumor immunity on account that they can produce proinflammatory cytokines that exert strong killing effects on invasive pathogens. M2 macrophages, also known as tumor-associated macrophages (TAMs), have anti-inflammatory and immunosuppressive activities, enhancing tumor metastasis and invasion [54].

In tumor tissues, a great quantity of TAMs has been observed. Several recent studies have shown that TDEs could induce the polarization of M2 macrophages. After treatment with exosomes derived from oral cancer cells, the expressions of CD206 and IL-10 in naive macrophages are increased, while there are no remarkable changes in the expressions of IL-1β and CXC10. Therefore, exosomes are indicated to promote the immunosuppressive M2 polarization of macrophages and subsequently enhance the proliferation and invasion of oral cancer cells [55,56]. Hsu et al. found that exosomes secreted from lung cancer cells under hypoxia promoted metastasis of lung cancer via the transfer of miR-103a [57]. Exosomal miR-103a secreted by tumor cells directly regulated the tensin homolog (PTEN) of macrophages. Once PTEN is downregulated, phosphatidylinositol 3-kinase/protein kinase B (PI3K/Akt) and signal transducers and activators of transcription 3 (STAT3) signaling pathways will be activated, resulting in increased accumulation of cancer-promoting cytokines, such as CCL2 and IL-10, and reduced antitumor immune response [58]. Aside from polarization, TDEs can modulate the M2 differentiation of monocytes. Exosomes derived from glioblastoma multiforme (GBM) are found to promote differentiation to alternative M2 phenotype macrophages [25].

The influence of TDEs on the functions of macrophages could modulate tumor growth, metastasis, and angiogenesis. Melanoma-derived exosomes are reported to induce hypoxia-inducible factor (HIF) activity in macrophages and promote neoangiogenesis in tumors [59]. In addition, by activating the nuclear factor kappa B (NF-κB) pathway, gastric cancer-derived exosomes induce the production of proinflammatory factors of macrophages, leading to enhanced proliferation, migration, and invasion of gastric cancer [26]. Other studies suggest that breast cancer-derived exosomes could increase the expression of Wnt5a in macrophages, and then transfer Wnt5a to tumor cells via exosomes to improve the invasive ability of tumor cells [27,28]. Furthermore, non-small-cell lung cancer-derived exosomes are found to be rich in miR-21 and miR-29a, which recruit macrophages and bind to TLR-8 to promote the secretion of IL-6 by immune cells, promoting the proliferation and migration of tumor cells [24].

### 3.3. Dendritic Cells

DCs, the major APCs in the immune system, act in initiating and maintaining T cell-mediated responses. The differentiation of DCs is mainly from both myeloid progenitors and lymphoid/monocytic cells. Conventional DCs (cDCs) and plasmacytoid DCs (pDCs) are the two main subtypes of human DCs [60]. After actively capturing, internalizing, and processing autologous antigens or foreign pathogenic antigens, DCs will activate T cells by virtue of stimulatory molecules and the MHC [61]. Recognition of foreign pathogenic antigens presented by DCs leads to the increased release of cytokines and expression of MHC and stimulatory molecules, indicating the dendritic cell maturation for the initiation of antitumor adaptive immune response. Meanwhile, inhibitory signals such as PD-L1 and FasL on DCs are also increased to prevent the overactivation of the immune system [62,63].

Studies have reported that TDEs can exert immunosuppressive effect by inhibiting differentiation of DCs. For example, TDEs can block the differentiation of a myeloid precursor and dendritic cell precursors so as to corrupt the myelopoiesis of cancer patients, resulting in reduction and accumulation in DCs and immunosuppressive MDSCs, respectively [64]. It is assumed that the differentiation of DCs can be inhibited by cyclooxygenase-1 and 2 (COX-1 and COX-2)-derived prostaglandin E2 (PEG2) [65]. Moreover, it is reported that TDEs induce MDSCs in a STAT-3-dependent manner. More data have shown that TDEs release a large amount of IL-6, HSP70, and HSP72, which are recognized as STAT-3 activators and inhibit the differentiation of DCs from MDSCs [29,30,31]. The expression of human leukocyte antigen G (HLA-G), the nonclassical MHC-I molecule, can be observed in various human tumors. HLA-G modulates the suppression of NK cells, DCs, and T cells by binding to inhibitory receptors. Moreover, HLA-G is also expressed on TDEs and has an inhibitory effect on the differentiation of DCs [32].

Apart from subverting DC biology by altering differentiation, TDEs also regulate the maturation of DCs. According to recent studies, TDEs with a couple of immunosuppressive biomolecules actively damage the maturation of DCs. It has been suggested that galectin-9 in TDEs may act as an important regulator of tumor progression. It could inhibit the maturation of DCs and also prevent antigen presentation from activating cytotoxic T cells in cerebrospinal fluid, resulting in the failure of antitumor immune response meditated by cytotoxic T cells [33]. The expression level of MHC-II on DCs is decreased by miRNA-212-3p in exosomes secreted by pancreatic cancer cells. MiR-212-3p reduces the level of regulatory factor X-associated protein (RFXAP), an important transcription factor for MHC-II. The decreased level of MHC molecules on DCs could be responsible for the compromised function of DCs [34]. Other studies have also found rich S100A9 on the exosomes from the lymphatic fluid of melanoma patients. TDEs carrying S100A9 downmodulate the expression of CD83, CD86, IL-12, and IL-15 on DCs [35]. Additionally, the level of glycolytic enzymes in TDEs is quite high. Glycolytic enzymes mainly convert extracellular glucose into ATP and restrain the maturation of DCs while promoting the expansion of MDSCs [66]. miRNA-203 in TDEs of pancreatic cancer cells downregulates TLR4 expression in DCs, leading to a decreased level of downstream TNF-α and IL-12, which inhibits the immunocompetence of DCs [15]. The presence of TDEs in the culture medium leads to the attenuation of costimulatory molecules and elicitation of inhibitory cytokines, such as TGF-β and PGE2, with a dose-dependent suppression of T cell proliferation and antitumor cytotoxicity [67,68]. Furthermore, HSP72 and HSP105 on the surface of TDEs induce the secretion of IL-6 by DCs in a TLR2- and TLR4-dependent manner. Then, IL-6, in turn, increases the level of MMP2, MMP9, and MMP13 to promote tumor cell invasion [36].

### 3.4. Natural Killer Cells

NK cells are a significant subset of lymphocytes and serve as critical effectors in antitumor immune response and immune surveillance. They can secret cytotoxic molecules or activate death receptors to destroy target cells [69]. Therefore, they usually work as the first line of defense to fight pathogens and tumors. A series of receptors regulate NK cell activity in specific ways. Important activating receptors include NKG2D, a C-type lectin-like receptor, and natural cytotoxicity receptors, such as NKP30, NKP46, and NPK44. NK cells are activated once these receptors recognize ligands on tumor cells or virus-infected cells [70].

With the purpose of contributing to immune escape, TDEs interfere with the amount, activity, and function of NK cells. According to studies on mice models, treatment with TDEs decreases the proportion of NK cells in the spleen and lung [71]. In tumor patients, NK cells present decreased activity and fewer activated receptors, such as NKp30 and NKG2D. NKG2D is the most critical receptor to stimulate the immune response of T cells. The level of NKG2D is decreased in NK cells in patients with head and neck cancer, acute myeloid leukemia, or melanoma treated with exosomes [72].

It is believed that TGF-β1 exerts a significant inhibitory effect on NK cells. Specifically, TGF-β, existing as TGF-LAP in TDEs, can be activated by integrins and reduce NKG2D expression through the phosphorylation of SMAD with the intention of inhibiting NK cell activation and cytotoxicity [73,74]. Besides, exosomes from primary clear renal cell carcinoma cells are found to be preferentially rich in TGF-β1 [75]. TDEs could also inhibit IL-2-induced stimulation of NK cells, downregulate the expression of cyclin D3, and inactivate the JAK-3 pathway [76]. Additionally, through miRNA profiling, high levels of miRNAs, such as miR-210 and miR-23a, are found in these hypoxic exosomes. MiR-23a has an immunosuppressive effect on NK cells by directly targeting CD107a [75].

### 3.5. Myeloid-Derived Suppressor Cells

MDSCs are composed of immature myeloid cells (IMCs) with strong immune suppressive ability in the TME. Under physiological conditions, IMCs differentiate into monocytes, DCs, and granulocytes in the bone marrow. However, under a pathological environment, such as cancer, the differentiation and maturation of IMCs are blocked, which leads to the expansion of MDSCs [77]. MDSCs are characterized by their capability in immune resistance and tumor development. Abundant suppressive molecules, such as reactive oxygen species (ROS), inducible nitric oxide synthase (iNOS), and PGE2, can be secreted by MDSCs to directly suppress the antitumor immunity dependent on effector T cells [78]. MDSCs can also induce the expansion of Tregs, promote the differentiation of Th17, or facilitate M2 macrophages to suppress immune responses [38].

TDEs have been reported to contribute to the production of immunosuppressive cell subsets by influencing the differentiation of the bone marrow. Xiang et al. first reported that bone marrow-derived cells (BMDCs) could uptake TDEs, which induce the differentiation of MDSCs by delivering PGE2 and TGF-β [40]. Other studies have also found that exosomes released by melanoma prohibit the differentiation of myeloid cells into DCs but induce their differentiation into TGF-β-secreting monocytic MDSCs, suppressing the proliferation and cytotoxic functions of T cells. Currently, it has been demonstrated that TDEs play significant roles in the expansion and functions of MDSCs [79]. Studies have found that exosomes from breast cancer cells support the expansion of early-stage MDSCs (eMDSCs) through activated JAK/STAT signaling regulated by miR-9 and miR-181a from exosomes [80,81]. Meanwhile, it is reported that tumor-derived soluble factors lead to the expansion of MDSCs by activating the ERK pathway. In addition, TDEs increase STAT-regulated Bcl-xL and Mcl-1 to prolong the survival of MDSCs [38,39].

TDEs also potentiate the immunosuppressive property of MDSCs. They could enhance the suppressive activity of MDSCs by boosting the release of nitric oxide. They could also trigger the suppressive function of MDSCs by promoting autocrine production of IL-6 in an HSP72/TLR2-dependent manner [30]. MDSCs driven by TDEs are reported to promote the formation of an immunosuppressive microenvironment by enhancing M2 polarization of monocytes and Th2 immune response [82]. In addition, exosomes derived from pancreatic ductal adenocarcinoma create an immunosuppressive background of myeloid cell via the transfer of SMAD4-associated miR-1260a and miR-494-3p [38,79].

## 4. Immunosuppressive Role of Exosomes Derived from Nontumor Cells

Nontumor cells in the tumor microenvironment also play important roles in the establishment of an immunosuppressive microenvironment. Exosomes secreted from these cells also inherit some functions of parental cells and participate in the regulation of antitumor immune resistance (Table 2).

### 4.1. Cancer-Associated Fibroblast-Derived Exosomes

Fibroblasts are cells of mesenchymal origin that produce extracellular matrix components to make up tumor stroma. Fibroblasts existing in cancer stroma, commonly known as cancer-associated fibroblasts (CAFs), participate in the formation of the TME [100]. Exosomes play important roles in the crosstalk between CAFs and cancer cells. Substantial reports have demonstrated that CAFs could transfer bidirectional signal into tumor cells through exosomes to regulate tumor development [101]. Exosomes secreted from breast tumor-derived CAFs promote the expression of PD-L1 in breast cancer cells by inhibiting the expression of large tumor suppressor kinase 2, which is related to the negative regulation of PD-L1 [83]. Upregulated PD-L1 on tumor cells suppresses the tumor-killing activity of T cells and NK cells and induces apoptosis of T cells.

### 4.2. Treg-Derived Exosomes

Most of the Tregs are immunosuppressive CD4 T cells that exert immunosuppressive functions in different ways. Recent studies have found that Tregs secrete more exosomes with membranous molecules compared with other T cell subtypes [102]. It has been demonstrated that Treg-derived exosomes play immunosuppressive roles in target cells by transmitting their immunosuppressive cargoes [84]. IL-35 has been demonstrated to be participated in suppressing the activation of T cells, and IL-35^+^ Tregs are enriched in tumors [103]. Tregs secrete IL-35 in response to the triggering of the T cell receptor to limit infiltration [103] and promote the exhaustion of T cells in the TME. Exosomal IL-35 is reported to target T and B cells to induce peripheral tolerance [84,85]. In addition, Treg-derived exosomes exert their suppressive functions via miRNAs to inhibit cell proliferation and cytokine release of Th1 cells [86]. Less secretion of IL-10 and IL-6 by DCs is induced by LPS stimulation with the treatment of exosomes enriched with miR-142-3p and miR-150-5 [87].

### 4.3. Myeloid-Derived Suppressor Cell-Derived Exosomes

Exosomes released by MDSCs inherit parental cell functions in immunosuppression, tumor growth, angiogenesis, invasion, and metastasis [94]. Multiple previous works have shown that the accumulation and expansion of MDSCs could be promoted by proteins and miRNAs carried in autocrine exosomes. The proinflammatory proteins S100A8 and S100A9, which are enriched in MDSC-derived exosomes, promote tumor growth and metastasis by mediating the accumulation and immunosuppressive functions of MDSC [88,89]. Additionally, HMGB1, a proinflammatory protein secreted by MDSCs, induces the production and accumulation of MDSCs. MiRNAs carried by MDSC-derived exosomes are also reported to regulate the suppressive functions of MDSCs due to their abilities to influence the differentiation and proliferation of myeloid cells [90,91]. MiRNA-155 enriched in exosomes causes the expansion of MDSCs by suppressing the level of SOCS-1 and increases the production of IL-10 [92,93]. Studies have also reported that MDSC-derived exosomes deplete CD8 T cells in vivo and inhibit the proliferation of CD8 T cells in vitro [94]. High levels of Fas and TNF-1α are found in MDSC-derived exosomes, triggering the apoptotic pathway, which suggests the potential role of exosomes in inducing apoptotic pathways [95]. Furthermore, treatment with MDSC-derived exosomes results in a substantial decline in M1 macrophages and an expansion of M2 macrophages [96].

### 4.4. Tumor-Associated Macrophage-Derived Exosomes

TAMs, which usually show an M2-like phenotype, play an important part in the TME. They are devoid of cytotoxic activity, provide growth factors for cancer cells, and have an immunosuppressive activity [54]. Several recent studies have shown that TAM-derived exosomes participate in the immune escape of tumor cells. Exosomes derived from M2 macrophages carry miR-155-5p, which induces immune escape and promotes the development of colon cancer by impairing the ZC3H12B-mediated stability of IL-6 [97]. Besides, exosomes enriched with miR-29a-3p mediate the FOXO3-AKT/GSK3β axis and improve the expression of PD-L1 in ovarian cancer cells, consequently promoting the proliferation and immune escape of ovarian cancer cells [98]. In addition, exosomes derived from M2 macrophages shuttle miR-21 to promote the survival, proliferation, migration, and invasion of glioma cells through decreased expression of paternally expressed gene 3 (PEG3). Bone marrow-derived macrophage-derived exosomes inhibit both the expansion and cytotoxicity of CD8 T cells to accelerate the immune escape of gliomas [99].

## 5. Exosomes as Cancer Biomarkers

**Figure 3:**
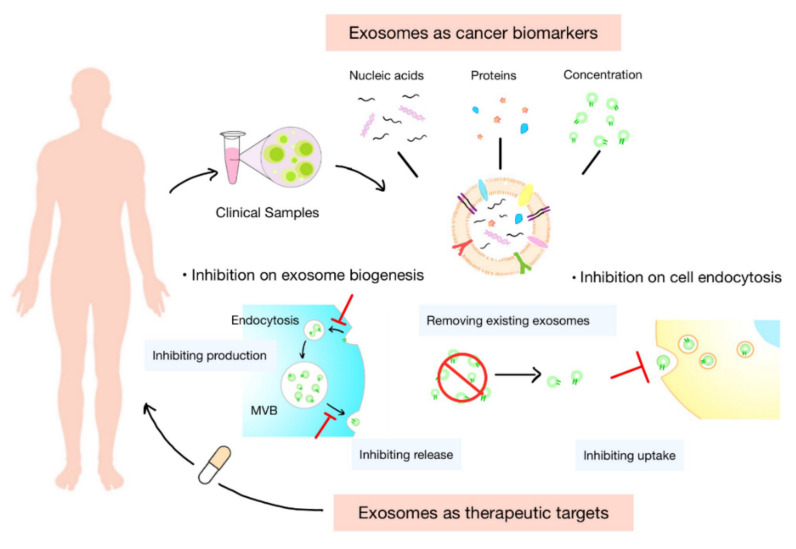
Clinical potential of exosomes. Exosomes normally serve as biomarkers or therapeutic targets in clinical settings. The concentration of exosomes and cargoes carried by them, such as nucleic acids and proteins, are potential diagnostic biomarkers of tumor and prognostic indicators of treatments. Inhibiting the biogenesis, release, or uptake of exosomes and removing the circulating cancer exosomes could be novel targets for anticancer therapies.

Exosomes are ideal substitutes for several biologically active molecules, such as proteins and transcripts. Cargoes carried by exosomes are inherited from their parental cells and partly reflect cell characteristics. They are protected by the membrane of exosomes from degradation during transportation. In addition, exosomes are widely distributed in various body fluids. These features of exosomes offer enormous potential for their use in the diagnosis and prognosis of tumor, as well as the prediction of therapeutic response (Figure 3).

### 5.1. Concentration of Exosomes

Exosomes are widely distributed to a variety of body fluids, including the blood, urine, saliva, cerebrospinal fluid [104]. Exosomes can serve as diagnostic indicators in “liquid biopsy” for various malignancies [105,106]. Numerous preclinical studies have verified the potential utility of exosomes in detecting and monitoring cancer. It is reported that the concentration of exosomes in the circulation increases in cancer patients (e.g., breast, ovarian, oral, and pancreatic cancer) [107]. Compared with the level of total exosomes, exosomes with specific markers are considered to be more accurate or sensitive for the diagnosis of tumor.

### 5.2. Nucleic Acids in Exosomes

Exosomes carry a large number of nucleic acids, such as mRNA, microRNAs (miRNAs), and long noncoding RNA (lncRNA), which can be used as biomarkers [108]. In addition, nucleic acids in exosomes can be amplified by PCR, guaranteeing the amount needed for detection [6]. There have been studies isolating exosomes from the plasma of glioma patients before and after treatment with vaccination. Analysis of 24 immunoregulatory genes in exosomes showed that the mRNA levels of four genes, consisting of IL-8, TGF-β, TIMP-1, and ZAP-70, are significantly downregulated after treatment. All of these genes are known to be associated with the clinical outcomes of glioma patients [109]. Specific exosomal mRNAs could also serve as diagnostic biomarkers for different tumors [110,111]. In addition, a lot of miRNAs are found to be potential diagnostic and prognostic biomarkers of tumor, such as miR-638 and miR-150-3p, which are downregulated in patients with hepatocellular cancer [112,113,114]. LncRNA in circulating exosomes have also been reported to be superior diagnostic biomarkers of tumor and be associated with the tumor progress and survival of patients [115,116].

### 5.3. Proteins in Exosomes

Exosomes inherit many origin-specific proteins from patient cells. The protein levels of circulating exosomes are reported to be increased in cancer patients and related to tumor grade, stage, treatment response, and survival rate of patients with different malignancies [117,118,119,120,121]. In addition, the contents of specific proteins in exosomes can also provide diagnostic or prognostic information. TGF-β1 in circulating exosomes is found to be biologically active and increased in patients with acute myeloid leukemia [122]. The high expression and high level of phosphorylation of MET, a hepatocyte growth factor receptor, are found to be increased in patients with advanced stages [6]. The level of circulating exosomal PD-L1 has been proved to predict response with immunotherapy in NSCLC patients [123]. All findings confirm the promise of exosomes as sensitive and specific biomarkers in the diagnosis and treatment of tumor.

### 5.4. Isolation and Identification of Exosomes

The isolation and purification of exosomes from biological samples are the basis of their detection. Ultracentrifugation, served as the most commonly used method, possesses significant advantages in the extraction of exosomes from a large number of samples. However, their disadvantages are also obvious: the long time consumption, special equipment, limited recovery, and low purity [124]. In addition to ultracentrifugation, a variety of separation methods based on the physical and chemical properties of exosomes have also been developed. The size-based isolation of exosomes includes exclusion chromatography and ultrafiltration. Compared with ultracentrifugation, ultrafiltration preserves the intact structures of exosomes with similar yield and purity [125]. On the other hand, exclusion chromatography guarantees the purity of exosomes with the efficient separation of proteins and lipids [126]. However, it also faces the challenges of low yield, consumed materials, and long time consumption. Precipitation achieves isolation of exosomes by binding with water molecules to accelerate the precipitate of exosomes. It is also trapped by the contamination of proteins and lipids [127]. The separation of exosomes dependent on immunoaffinity possesses the highest specificity with a simple operation. Through antibodies and aptamers targeting surface proteins on exosomes, exosomes with high expression of specific proteins are immobilized on various carriers, such as magnetic and latex beads, and isolated by magnetic and centrifugal forces, respectively [128]. However, the isolated exosomes are only a small group of exosomes, and the binding sites of the targeted proteins may be blocked, which interferes with the function or detection of the proteins. At present, the detection and quantification of exosomes are mainly focused on the proteins in exosomes. Western blotting, flow cytometry, and ELISA are the most commonly used methods to quantify the protein levels in exosomes by virtue of antibodies. All of these methods possess limited detection limits from ng to pg and complex operations that impede the real-time monitoring of exosomes [129]. In recent years, detection methods targeting nucleic acids and lipids carried by exosomes are constantly emerging, which expands the clinical indicators and contributes to the monitoring of the total population of exosomes [130]. New techniques, such as surface plasmon resonance (SPR) and Raman tweezers microspectroscopy (RTM), have also been introduced in the detection of exosomes [130,131]. Benefiting from the development of microfluidic chip technology, innovative methods with low demand for sample volume, high recovery, and short time consumption are established [132]. They can complete the simultaneous isolation and detection of exosomes in one chip.

## 6. Exosomes as Therapeutic Targets

Cells in the TME constantly release exosomes into the surrounding tumor environment and circulation. These exosomes play pivotal roles in tumor immune escape and immune therapeutic resistance in vivo. Therapeutic approaches that rely on modulating the level or function of exosomes may help address the above-mentioned problems [133]. At present, the regulation of exosomes in vivo mainly focuses on inhibiting their secretion or blocking their interaction with cells. Moreover, some studies try to promote the clearance of exosomes from circulation (Figure 3).

### 6.1. Inhibition of Biogenesis of Exosomes

The inhibition of exosome biogenesis is an essential step when targeting the regulation of exosome in circulation. Due to the complicated nature of exosome biogenesis, developing inhibitors that effectively and specially block this process remains a challenge. Inhibitors of ESCRT-dependent transportation of exosomes have been found in recent years, including manumycin A, tipifarnib, and sulfisoxazole. Manumycin A mainly diminish the production level of exosomes by inhibiting the Ras/Raf/ERK1/2 pathway and the expression of hnRNPH1 to attenuate exosome biogenesis and secretion. Manumycin A also inhibits the activity of neutral sphingomyelinase 2 (nSMase2). About 50–65% of exosome biogenesis is inhibited by 250 M manumycin A in different cancer cell lines [134]. Tipifarnib reduces the production level of exosomes by inhibiting the phosphorylation of ERK, which is also necessary for tumor growth. An amount of 1 µM of tipifarnib could inhibit exosome biogenesis by 70% in the prostate cancer cell line C4-2B [135]. Sulfisoxazole is an antibacterial drug that performs inhibition on the secretion of exosomes by targeting ESCRT- or Rab-related proteins in breast adenocarcinoma cell lines [136]. In addition, exosome biogenesis inhibition can generally occur through inhibition on the release of exosomes. GW4869 is one of the most widely used noncompetitive inhibitors of nSMase, which is important for the membrane invagination of MVB [137]. In a previous study, the secretion of vesicles with a size range of 100–200 nm was decreased in breast cancer cells treated with GW4869 [138].

### 6.2. Inhibition of Endocytosis of Exosomes by Recipient Cells

Another theoretical approach is to block interactions between cells and exosomes. However, the unclear mechanism of exosome trafficking and its target definition limit this approach. It is pointed out that both exosomes and the recipient cells determine their interactions [139]. Dynasore is a widely used highly efficient noncompetitive inhibitor of GTPase activities [140]. It exerts an inhibitory effect on the production of clathrin-coated endocytic vesicle [141]. Another inhibitor targeting exosome uptake is heparin. The co-localization of heparin and exosomes is found under microscopy [142]. It is found to completely interact with cell-surface heparan sulfate proteoglycans (HSPGs) and interfere with the transfer of brain tumor cell-derived exosomes into recipient cells [143].

## 7. Conclusions

This review systematically summarized the immunosuppressive roles of both tumor-cell- and non-tumor-cell-derived exosomes in the TME. Various tumor processes, including growth, invasion, metastasis, angiogenesis, and immunotherapeutic resistance, are under the modulation of exosomes. In the TME, exosomes play critical roles in orchestrating an immunosuppressive microenvironment favorable for tumor development. It is essential to understand the correlations between exosomes and immune suppression for the explorations on diagnostic and prognostic indicators of tumor and the design of more effective antitumor immunotherapies. A comprehensive disclosure of the above mechanisms contributes to the development of a personalized targeted immunotherapy, improvement of the therapeutic effects, and reduced possibility of potential adverse reactions. However, there are still many unsolved problems, such as the different influences on the same immune cells from exosomes derived from different cells and the different roles of exosomes in different microenvironments. The complex functions of exosomes are inextricably linked to the diverse populations of exosomes, also known as heterogeneity of exosomes. The heterogeneity causes variation of the molecular profiles of single subgroups of exosomes. Due to their heterogeneity, bulk analysis of total exosomes is insufficient to accurately identify the disease state. Thus, the single-particle assay that distinguishes their cellular origin, size, content, and functional impact on recipient cells is the future direction of the detection of exosomes. However, the current isolation and quantification of specific subpopulations of exosomes, currently mainly based on affinity, are still far away from clinical application. How to capture exosomes noninvasively, efficiently, and simply and detect exosomes in an accurate and sensitive way should be addressed properly. In addition, there is also an urgent demand for the elimination or functional blockade of specific subgroups of exosomes.

## Figures and Tables

**Table 1:** Immunosuppressive role of tumor cell-derived exosomes.

Target Cells	Functional Molecules	Effects	References
T cells	Galectin-9	Apoptosis of EBV-specific CD4 T cells and inhibition on Th1 cell function	[14]
	FasL	Apoptosis of T cells	[15]
	PD-L1	Inhibited proliferation and functions of T cells	[16]
	ULBP/MICA	Inhibition on the NKG2D signaling pathway	[17]
	TGF-β	Downregulation of NKG2D on CD8 T cells	[18]
	MiRNAs	Inhibition on the differentiation of Th cells	[19]
Tregs	TGF-β	Upregulation of Treg-related genes	[20]
	IL-10/IL-2	Increased amount and enhanced function of Tregs	[21]
Bregs	——	Differentiation into TGF-β-producing Bregs	[22]
	HMGB1	Increased TIM-1 Breg cells	[23]
Macrophages	miRNAs	Promotion on the IL-6 secretion of immune cells	[24]
	CSPG4, EGFR, and integrins	Increased M2 macrophages	[25]
	——	Increased proinflammatory factors	[26]
	Wnt5a	Improvement on the invasion ability of tumor	[27,28]
DCs	IL-6, HSP70, and HSP72	Activation of STAT3	[29,30,31]
	HLA-G	Suppression on T cells, NK cells, and DCs	[32]
	Galectin-9	Inhibition on the maturation and failure of activating cytotoxic T cells	[33]
	miRNAs	Inhibited RFXAP and toll-like receptor 4 (TLR4) expression in DCs	[34]
	S100A9	Decreased costimulatory molecules on DCs	[35]
	HSP72 and HSP105	Increased secretion of IL-6 of DCs	[36]
NK cells	TGF-β	Downregulation of NKG2D and reduced cytolytic activity of NK cells	[37]
MDSCs	miRNAs	Activation of STAT1 and STAT3 pathways and expansion of MDSCs	[38,39]
	——	Release of NO from MDSCs	[15]
	HSP72/TLR2	Autocrine production of IL-6	[30]
	PGE2 and TGF-β	Differentiation of MDSCs from myeloid cells	[40]

**Table 2:** Immunosuppressive effects of exosomes secreted by nontumor cells.

Origin of Exosomes	Target Cells	Functional Molecules	Mechanisms	References
Cancer-associated fibroblasts	PD-L1	miR-92	Promote YAP1 nuclear translocation and increase PD-L1 transcription	[83]
Tregs	T cells and B cells	IL-35	Induce peripheral tolerance of T cells and B cells by transferring IL-35	[84,85]
	T cells and DCs	miRNAs	Inhibit proliferation and cytokine release of Th1 cells and DCs	[86,87]
MDSCs	MDSCs	S100A8 and S100A9	Mediate accumulation and immunosuppressive function of MDSCs	[88,89]
	MDSCs	miRNAs	Promote expansion, differentiation survival, and IL-10 production of MDSCs	[90,91,92,93]
	T cells	——	Induce apoptosis of T cells	[94,95]
	Macrophages	——	Induce decline of M1 macrophages and expansion of M2 macrophages	[96]
Tumor-associated macrophages	——	miR-155-5p	Impair stability of IL-6	[97]
	Ovarian cancer cells	miR-29a-3p	Increase expression of PD-L1	[98]
	T cells	——	Inhibit cell cytotoxicity of CD8 T cells	[99]

## Data Availability

Not applicable.
